# Case report: Novel treatment regimen for *enterovirus* encephalitis in SCID

**DOI:** 10.3389/fimmu.2022.930031

**Published:** 2022-09-13

**Authors:** Kritika Chetty, Iek Cheng, Marios Kaliakatsos, Luis Ignacio Gonzalez-Granado, Dimitra Klapsa, Javier Martin, Alasdair Bamford, Judith Breuer, Claire Booth

**Affiliations:** ^1^Department of Immunology, Great Ormond Street Hospital, London, United Kingdom; ^2^Molecular and Cellular Immunology Section, Great Ormond Street Institute of Child Health, University College London, London, United Kingdom; ^3^Pharmacy department, Great Ormond Street Hospital for Children National Health Service (NHS) Foundation Trust, London, United Kingdom; ^4^UCL Faulty of Infection, Immunity and Inflammation, UCL Great Ormond Street Institute of Child Health, London, United Kingdom; ^5^Department of Neurology, Great Ormond Street Hospital, London, United Kingdom; ^6^Servicio de Pediatria, Hospital Universitario 12 de octubre, Madrid, Spain; ^7^Facultad de Medicina, Universidad Complutense de Madrid, Madrid, Spain; ^8^Vaccines Division, The Medicines and Healthcare products Regulatory Agency, Potters Bar, United Kingdom; ^9^Department of Infectious Diseases, Great Ormond Street Hospital for Children, London, United Kingdom; ^10^Infection, Immunity and Inflammation Teaching and Research Department, Great Ormond Street Institute of Child Health, Faculty of Population Health Sciences, University College London, London, United Kingdom; ^11^Division of Infection and Immunity, University College London, London, United Kingdom

**Keywords:** case report, primary immunodeficiency diseases, *enterovirus*, infectious encephalitis, antiviral agents, severe combined immunodeficiency, gene therapy (GT)

## Abstract

Most non-polio *enterovirus* infections in immunocompetent individuals are acute and self-limiting in nature; however, infection can be severe, chronic and have devastating outcomes in immunocompromised hosts. Therapeutic strategies have predominantly involved supportive care, with the lack of approved antiviral treatments proving challenging for management. We report a case of an 8-month-old child who presented with severe *enterovirus* encephalitis following gene therapy for X-linked severe combined immunodeficiency (X-SCID) and who demonstrated clinical and microbiological improvement after a novel regimen of favipiravir, fluoxetine, and high-dose intravenous immunoglobulin (IVIg). The patient presented 6 weeks post–gene therapy with rapid neurological deterioration in the context of incomplete immune reconstitution, with microbiological and radiological evidence confirming *enterovirus* encephalitis. His neurologic examination stabilised 8 weeks after treatment, and he subsequently demonstrated excellent immune recovery. This is the first case report of combined therapy with favipiravir, fluoxetine, and high-dose IVIg in the context of severe *enterovirus* encephalitis in an immunocompromised host. This case highlights the importance of considering *enterovirus* encephalitis in immunocompromised patients presenting with both acute and chronic neurological signs, as well as developmental regression. The demonstrated treatment success and the associated low risk of toxicity warrant further investigation of this therapeutic regimen.

## Introduction

*Enterovirus*es are small RNA viruses from the picornaviridae family, a genus of 12 species of which seven infect humans, ranging from *Enterovirus* A, B, C, or D and Rhinovirus A, B, and C. While most non-polio *enterovirus* infections in immunocompetent individuals are self-limiting, infections in immunocompromised hosts can become chronic and severe.

There is a lack of approved antiviral treatments for *enterovirus* infections that presents a particular challenge in immunocompromised individuals. We report an 8-month-old child who presented with *enterovirus* encephalitis 6 weeks post–gene therapy for X-linked severe combined immunodeficiency (X-SCID), with clinical and microbiological improvement after a novel regimen of favipiravir, fluoxetine, and high-dose intravenous immunoglobulin (IVIg).

## Case report

The child was born at term to non-consanguineous parents with an uneventful neonatal period. He developed recurrent bacterial infections from 1 month of life, with omphalitis and bilateral ear and prepucial discharge, positive for *Stenotrophomonas maltophilia* and *Streptococcus pyogenes*. He had an 8-week history of cough and was found to be Polymerase Chain Reaction (PCR) positive for rhinovirus on nasopharyngeal aspirate. His infectious history, in addition to a skin rash and eosinophilia suggesting possible Omenn syndrome, prompted investigation for primary immunodeficiency. There was no significant family history of recurrent infections or diagnosed inborn errors of immunity. His total lymphocyte count was normal (3.78 × 10^9^/L); however, he had marked T and NK cell lymphopaenia with 50/µl CD3+ T cells, 3,660/µl CD19+ B cells, 20/µl CD16+CD56+ Natural Killer cells, 40/µl CD4+ T cells, undetectable naive T cells, and an absent Phytohaemagglutinin (PHA) response Natural Killer (**Table 1**). There was no maternal engraftment, and viral screen was negative. Sanger sequencing demonstrated a pathogenic hemizygous c.455T>G variant in the *IL2RG* gene, denoting the amino acid valine was changed to glycine at residue 152, consistent with X-SCID. He was referred for gene therapy, as no matched related donor was available for haematopoietic stem cell transplant (HSCT). Incidentally, he developed a self-resolving idiopathic facial palsy at 3 months of age, with normal MRI-Brain.

**Table 1:** Relevant laboratory parameters pre- and at various time points post-IMP.

Months post-Investigational Medicinal Product (IMP)	Pre-Investigational Medicinal Product (IMP)	0–1	1–2	2–3	3–4	6	Units
**Immunology**							
Lymphocyte count	3,780	3,120	4,510	6,550		4,070	cells/µl
CD3+ T Cells	50	40	40	800		2,840	cells/µl
CD19+ B Cells	3,660	2,100	4,090	5,470		1,030	cells/µl
CD16+56+ NK Cells	20	820	290	160		90	cells/µl
CD3+CD4+ T cells	40	10	20	630		1,410	cells/µl
CD3+CD8+ T cells	10	0	0	70		390	cells/µl
Naive CD4+ T cells	Undet	Undet	Undet	630		2,410	cells/µl
Naive CD8+ T cells	Undet	Undet	Undet	70		390	cells/µl
PHA Stimulation	Absent						
Common gamma chain (γc) expression	Equivalent to control						
Signal transducer and activator of transcription 5 (STAT5) Phosphorylation	Absent in response to Interleukin 2 (IL-2); Interleukin 7 (IL-7); Interleukin 15 (IL-15)						
Maternal engraftment	Nil						
**Microbiology**							
Epstein-Barr Virus; CMV = Cytomegalovirus (EBV), Cytomegalovirus (CMV), Adenovirus PCR (Blood)	Negative	Negative	Negative	Negative			
Respiratory Viral Panel^a^ PCR				Rhinovirus			
Diagcore Respiratory Panel^b^ PCR	Rhinovirus/enterovirus: cycle threshold (cT) 22.0						
Stool Microbiology^c^ (Culture & PCR)	Negative	Negative	Negative				
CSF Microbiology^d^ (Culture & PCR)			Negative				
**Enterovirus PCR**							
NPA	Equivocal: cycle threshold (cT) 38.44			cycle threshold (cT) 27.42	Negative		
CSF				cycle threshold (cT) 35.18	Negative	Negative	
Blood				Negative	Negative		
Stool			cycle threshold (cT) 35.11	cycle threshold (cT) 34.93			
**CSF–Other Parameters**							
CSF White Blood Cell (WBC)			23	24	10	6	× 10^6^/L
CSF Red Blood Cell (RBC)			4	<1	<1	<1	× 10^6^/L
CSF Glucose			2.3	2.4		2.7	mmol/L
CSF Lactate			1.6			1.5	mmol/L
CSF Protein			1.06	0.75	0.57	0.3	g/L
CSF IL-6			105	47.81	25		pg/ml

**^a^** = Coronavirus NL63, Coronavirus 229E, Coronavirus OC43, Coronavirus HKU1, Rhinovirus, Enterovirus.

**^b^** = Influenza A, Influenza B, Influenza A H1N1, Coronavirus 229E, Coronavirus OC43, Coronavirus NL63, Coronavirus HKU1, Parainfluenza 1, Parainfluenza 2, Parainfluenza 3, Parainfluenza 4, Influenza A H3, Rhino/Entero, Adenovirus, Respiratory Syncytial Virus (RSV) A/B, Human metapneumovirus (HMV) A/B, Bocavirus, Mycoplasma pneumoniae, Legionella pneumoniae, Bordetella pertussis, Corona COVID-19.

**^c^** = Norovirus G1, Norovirus G2, Rotavirus, Adenovirus, Astrovirus, Sapovirus.

**^d^** = CMV, adenovirus, Herpes Simplex Virus (HSV)1, Herpes Simplex Virus (HSV)2, Varicella Zoster Virus (VZV), Parechovirus, Parvovirus, Astrovirus, 16s, 18s, Toxoplasma.

Undet = undetected.

At 6 months old, he underwent lentiviral gene therapy with low-dose busulfan conditioning. Prior to mobilisation and harvest of haematopoietic stem cells, he received a short course of oral steroids to treat an underlying inflammation, as suggested by eczematous skin rash, thrombophilia, and eosinophilia. This improved his symptoms. He was discharged 3 weeks following cell infusion.

**Figure 1:**
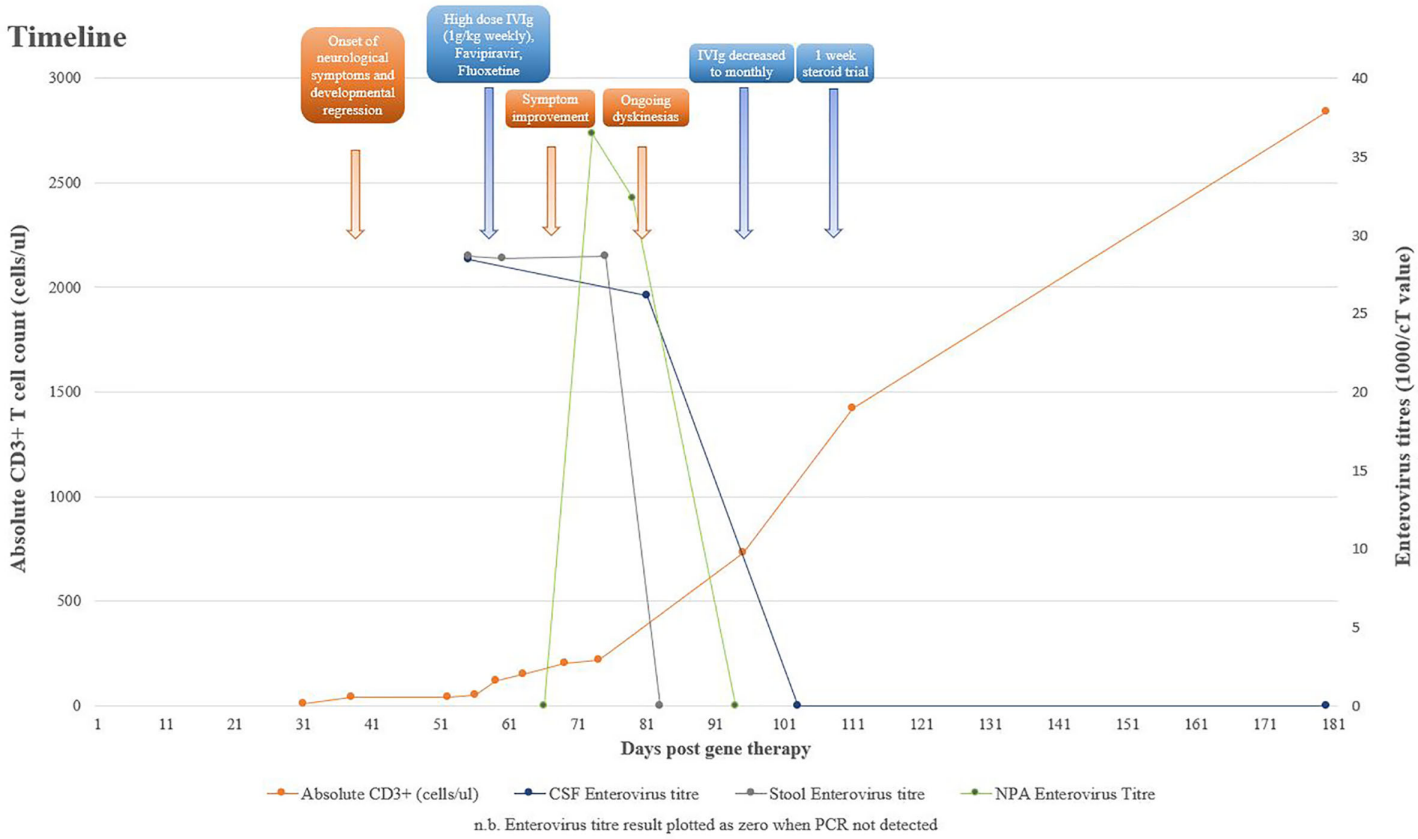
Timeline of clinical symptoms with relevant laboratory parameters and treatments.

At 6 weeks post–gene therapy, he developed rapid neurological deterioration with bilateral squint, inability to fix and follow, upgazing episodes, limb dyskinesia and dystonic movements, truncal hypotonia, and encephalopathy (**Figure 1**). His symptoms developed while on a prophylactic antimicrobial regimen of co-trimoxazole, phenoxymethylpenicillin, ciprofloxacin, acyclovir, immunoglobulin replacement, and itraconazole. He had no fevers or other infective symptoms, rashes, feeding issues, or gut symptoms. Cardiovascular, respiratory, and abdominal examinations were unremarkable, and there was no lymphadenopathy. Immune recovery was emerging with 40/µl CD3+ T cells, 20/µl CD4+ T cells, and undetectable naive T cells. Lumbar puncture demonstrated a raised white cell count (WCC) of 23 × 10^6^/L, protein of 1.06 g/L, glucose of 2.3 mmol/L, and elevated Interleukin 6 level at 105 pg/ml. Cerebrospinal fluid (CSF) *Enterovirus* PCR was positive with a cT value of 35.18, subsequently identified by deep sequencing as Coxsackievirus B1. Blood PCR was negative for *enterovirus*; however, both stool and Nasopharyngeal aspirate (NPA) were positive with cT values of 34.9 and 27.4, respectively. Retrospective analysis of an NPA sample prior to gene therapy was equivocal for *enterovirus* with a cT value of 38.44.

**Figure 2:**
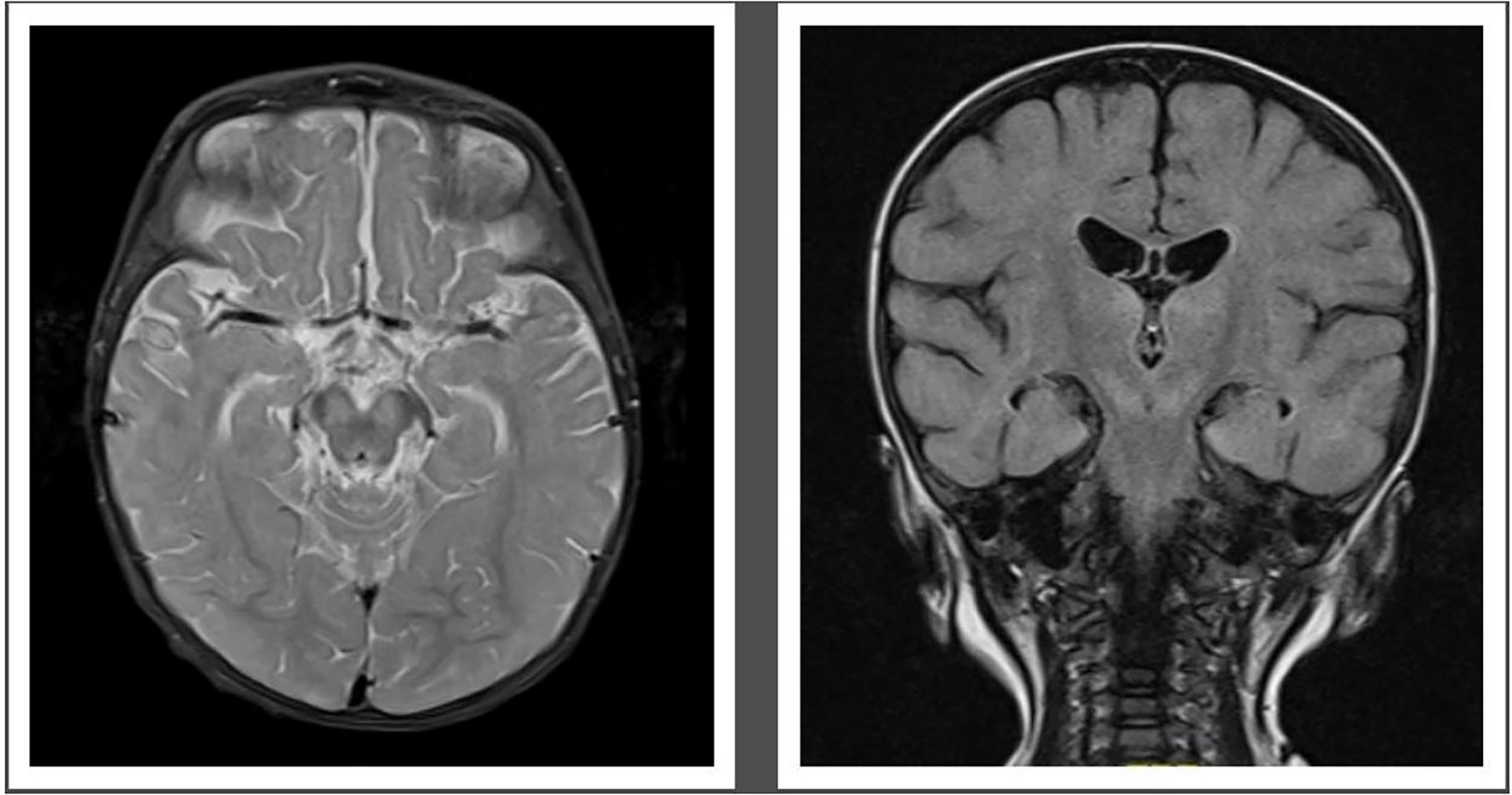
Pretreatment MRI-Brain demonstrating T2 hyperintense signal in midbrain (left) and hyperintense Fluid attenuated inversion recovery (FLAIR) signal in both thalami and midbrain (right).

MRI demonstrated abnormal T2 signal in thalami and brainstem with some restricted diffusion suggestive of viral encephalitis (**Figure 2**). He commenced treatment with IVIg 1 g/kg weekly, favipiravir, and fluoxetine after receiving approval from the hospital Drugs and Therapeutics Committee (DTC). Oral favipiravir was dosed initially at 500 mg/500 mg/200 mg 8 hourly on day 1, followed by 200 mg three times a day thereafter. Oral fluoxetine was initiated at a low dose of 2.5 mg daily and titrated to a maximum dose of 0.75 mg/kg/day. Treatment was associated with clinical improvement, with resolution of encephalopathy, improvement in limb dystonia, and no further paroxysmal eye movements.

His neurological progress plateaued 4 weeks after initiation of therapy, with intermittent dystonia and an evolving hypertonia. His developmental regression was ongoing, having not yet regained independent sitting, no longer reaching purposefully for objects, or vocalising or smiling as previously. However, he was felt to have an overall improvement in his neurologic examination since the beginning of his admission, particularly his eye movement disorder, and he was demonstrating better head control. IVIg frequency was reduced from weekly to monthly, as previously planned, and gabapentin and clonidine commenced for limb hypertonia. A repeat CSF at this point demonstrated an equivocal result for *enterovirus* PCR with a cT of 38.22, a WCC of 24 × 10^6^/L, protein of 0.75 g/L, and glucose of 2.4 mmol/L. At 6 weeks after therapy initiation, the CSF was negative for *enterovirus*, with a reducing WCC of 10 × 10^6^/L and protein of 0.57 g/L.

He experienced significant ongoing dyskinesias despite decreasing and ultimately negative *enterovirus* loads in the CSF and repeat MRI not suggestive of ongoing inflammation. It was hypothesised that he was developing an emerging movement disorder on a background of *enterovirus* encephalitis that significantly affected the basal ganglia, which was expected to become more pronounced over time. It was difficult to determine based on clinical assessment whether an ongoing neuroinflammatory process was contributing to his evolving dyskinesias and hypertonia, and given the recent sharp rise in CD3+ T cells, to 800 cells/µl, a 1-week trial of 2-mg/kg prednisolone was undertaken to treat possible immune reconstitution inflammatory syndrome. This did not significantly improve his movement disorder.

Virus recovered from the CSF was found to be sensitive in hep2 cell plaque reduction assays to fluoxetine hydrochloride, demonstrating complete inhibition at 25 µM. While *enterovirus*es have shown susceptibility to favipiravir, pocapavir, and ribavirin, all three drugs were toxic to cells, preventing sensitivity testing of this isolate (1–3). The case was discussed in multidisciplinary team (MDT) fora, including a combined Neurology and Infectious Diseases MDT meeting, which provided consensus support for the novel approach to management of such complex cases. Combination therapy was continued despite lack of *in vitro* evidence of sensitivity to favipiravir in view of a clinical response.

After 8 weeks of therapy, his neurological signs stabilised, with some ongoing dystonic posturing, but no prolonged dystonic events. He had excellent immune recovery with 1,420/µl CD3+ cells, 1,190/µl CD4+ T cells, normal naive T cells, PHA and gamma chain expression, and expected levels of gene marking. He was discharged 4 months after gene therapy. At the last follow-up at 25 months of age, he is able to sit with support, is reaching out for toys with both hands, is smiling and laughing, can fix and follow, has normal hearing, is making vocalisations (vowel sounds), and is fed orally. He has asymmetrically increased 4-limb tone and has ongoing rehabilitation.

## Discussion

This is the first case report of combined therapy with favipiravir, fluoxetine, and high-dose IVIg in the context of severe *enterovirus* encephalitis in an immunocompromised host. The lack of any approved antiviral treatments for *enterovirus* infection presents a particular therapeutic challenge in immunocompromised individuals. The use of high-dose IVIg in chronic enteroviral meningitis has previously been documented, as has fluoxetine as an adjunct to high-dose IVIg in a patient with X-linked agammaglobulinaemia (2, 4). Favipiravir has shown *in vitro* activity against *enterovirus*es; however, to our knowledge, there have been no reports of its use in enteroviral Central nervous system (CNS) infection (5).

Favipiravir is a novel antiviral compound that was developed in 2002 as an inhibitor of influenza virus replication (5). It works by selectively and potently inhibiting the RNA-dependent RNA polymerase (RdRP) of RNA viruses, inducing mutagenesis and resulting in a loss of viral fitness. RdRP domains are not present in human cells, however, are conserved among RNA viruses, and when assessed against a panel of *enterovirus*-D68 strains, they proved to be a weak inhibitor of *in vitro* replication of *enterovirus* (3). Previous *in vitro* assessment confirmed the mechanism of action of favipiravir by demonstrating that an S121N mutation in the finger subdomain of the 3D polymerase of the EV-A71 strain of *enterovirus* conferred resistance to favipiravir (6). Low levels of the active metabolite have been detected in the brain; however, assessment of this is limited and has only been conducted in mice (7). The drug is approved in Japan for treatment of non-complicated infections with influenza virus; however, it is also active against other RNA viruses including Ebola virus. Mixed evidence of clinical benefit exists in coronavirus disease 2019 (COVID-19), with shorter times to clinical improvement; however, there was no significant difference in mortality in two meta-analyses comparing treatment with and without favipiravir in patients with COVID-19 (8, 9). Additionally, a spectrum of difference in adverse events was seen between the two groups, ranging from both more and less adverse events in the favipiravir group compared to the control to no difference between the favipiravir and control group.

Treatment for this patient required approval from the hospital DTC, as favipiravir is unlicensed in the United Kingdom, where treatment took place. The drug was imported from Japan where it is licensed for influenza A and B in adults. A lack of availability of pocapavir, toxicity associated with ribavirin, and a cumulative experience at our tertiary centre in using favipiravir in RNA virus infections in immunocompromised hosts favoured the selection of favipiravir in this treatment regimen. Dosing has previously been established for adult Japanese patients infected with influenza and body weight-based dosing proposed in a trial in children infected with Ebola, which informed the dosing in this patient (10, 11). The drug holds a favourable safety profile, with the most frequently reported adverse events including mild to moderate diarrhoea and a decrease in neutrophil count. No adverse effects were reported in this patient, with monitoring throughout treatment *via* regular physical assessments screening for rash and gastrointestinal side effects and measurement of liver function tests, full blood counts, and electrolytes, all of which were within normal range (12).

In immunocompetent individuals, 90% of non-polio *enterovirus* infections are asymptomatic or mild febrile illnesses. Infection can also manifest more severe disease, for example, viral meningitis, accounting for between 48% and 95% of cases where a causative virus is identified (13). Conversely, infections in immunocompromised hosts including primary immunodeficiency, neonates, and post-HSCT result in more serious disease often of a chronic nature ranging from meningoencephalitis, dermatomyositis, hepatitis, and/or arthritis (14). Agammaglobulinaemic patients can present with developmental regression and ataxia (15). The course of disease in the immunocompromised host tends to be slow and progressive with periods of remission and relapse. Meningoencephalitis specifically features a range of neurological symptoms including ataxia, loss of cognitive skills, and paraesthesias (16).

Immune reconstitution inflammatory syndrome (IRIS) describes an excessive inflammatory response to infection occurring during immune reconstitution after a period of immunosuppression. The history of self-limiting facial palsy may have been indicative of *enterovirus* infection, with the subsequent neurological symptoms being a manifestation of IRIS. However, a short course of corticosteroids, the most frequently used agent for the treatment of IRIS, did not result in resolution of symptoms.

Most transplant centres do not routinely screen for *enterovirus* infection pretransplant or posttransplant. Given the devastating effects of infection in the immunocompromised, as highlighted here, it raises the question of whether screening for *enterovirus* should become a standard of care. Without any definitive treatments or approved antiviral therapies for *enterovirus*, screening may not provide any significant additional benefit to morbidity and mortality. However, prompt initiation of novel treatments in post-HSCT and gene therapy infections may mitigate neurological deterioration and control the virus before recovery of immune function.

Besides the possibility that this novel therapeutic regimen effectively treated this patient’s *enterovirus* infection, it is conceivable that this child’s improvement may have been due to immune recovery over time. While there is a lack of literature on the natural history of *enterovirus* in X-SCID patients treated with gene therapy, an evaluation of 64 T cell-depleted adult allograft recipients found no correlation between immune recovery and the occurrence of *enterovirus* infections, in particular, no difference in the median estimate of CD4+ T-cell count and absolute lymphocyte count posttransplant in those with and without *enterovirus* infections (17).

Given that the course of IRIS is to improve over time, despite the lack of immediate symptom improvement with steroids, IRIS may also be another explanation for the constellation of neurological symptoms seen secondary to the infective insult. It is extremely challenging to determine the contribution of infection and inflammation in the context of IRIS and therefore whether anti-infectives or anti-inflammatories are to be prioritised. Given the diagnostic ambiguity, often, both will be required.

Although it is difficult to objectively assess the family’s satisfaction with this novel antiviral treatment regimen, given the neurologic sequelae of this patient’s disease, the family was happy to be offered this new treatment and was informed and satisfied to continue with the treatment regimen.

This case highlights the importance of considering *enterovirus* encephalitis in immunocompromised patients presenting with both acute and chronic neurological signs, as well as developmental regression. The novel therapy regimen of favipiravir, fluoxetine, and high-dose IVIg was associated with neurological improvement and, given the low risk of toxicity, is worth consideration on a case-by-case basis and warrants further investigation (**2**).

## Data availability statement

The original contributions presented in the study are included in the article/supplementary material. Further inquiries can be directed to the corresponding author.

## Ethics statement

Written informed consent was obtained from the minor(s)’ legal guardian/next of kin for the publication of any potentially identifiable images or data included in this article.

## Author contributions

KC and CB: preparing manuscript. KC, IC, MK, LG-G, AB, JB, CB: patient management, providing diagnostic and treatment results. DK, JM: providing diagnostic and treatment results. All authors contributed to the article and approved the submitted version.

## Funding

All research at Great Ormond Street Hospital NHS Foundation Trust and UCL Great Ormond Street Institute of Child Health is made possible by the NIHR Great Ormond Street Hospital Biomedical Research Centre. The views expressed are those of the author(s) and not necessarily those of the NHS, the NIHR or the Department of Health. JB is supported by the Wellcome Trust. The funders had no role in study design, data collection and analysis, decision to publish, or preparation of the manuscript.

## Conflict of interest

The authors declare that the research was conducted in the absence of any commercial or financial relationships that could be construed as a potential conflict of interest.

## Publisher’s note

All claims expressed in this article are solely those of the authors and do not necessarily represent those of their affiliated organizations, or those of the publisher, the editors and the reviewers. Any product that may be evaluated in this article, or claim that may be made by its manufacturer, is not guaranteed or endorsed by the publisher.
